# Effect of Bushen Yutai Recipe on IVF Patients Subjected to Mild Ovarian Stimulation

**DOI:** 10.3389/fmed.2020.541537

**Published:** 2020-11-12

**Authors:** Xiaomei Jiang, Hua Yan, Xiufang Zhong, Guoqing Tong, Wuwen Zhang

**Affiliations:** ^1^Department of Urology and Reproductive Medicine, Seventh People's Hospital Affiliated to Shanghai University of Traditional Chinese Medicine, Shanghai, China; ^2^Department of Reproductive Center, Shuguang Hospital Affiliated to Shanghai University of Traditional Chinese Medicine, Shanghai, China

**Keywords:** endometrial receptivity, herbal, mild ovarian stimulation, traditional chinese medicine, *in vitro* fertilization and embryo transfer, herbal

## Abstract

This article investigated the effects of the traditional Chinese medicine (TCM) herbal recipe, Bushen Yutai, on *in vitro* fertilization (IVF) patients subjected to mild ovarian stimulation. Two hundred nineteen infertile patients were randomly divided into 2 groups: the control group and herbal treatment group. By studying, we found estrogen levels (E_2_) on the human chorionic gonadotropin (hCG) triggering day were significantly lower in the control group (*P* < 0.05), with positive blood flow being less detected by ultrasound scanning on both the day of hCG triggering and day of fresh embryo transfer for the control group (*P* < 0.05). Additionally, the blood flow index, retroactive and proactive inhibition, was higher in the control group, whereas the fertilization rate and number of high-quality embryos in the control group were lower than the control TCM experimental group (*P* < 0.01). The expression levels of the endometrial receptivity gene, vascular endothelial growth factor (VEGF), were lower in the control group vs. the TCM experimental group on the day of fresh embryo transfer (*P* < 0.05), whereas the rate of fresh embryo transfer in the control group was lower than the TCM experimental group (*P* < 0.05). In conclusion, the TCM could increase the E_2_ during the IVF stage, with a higher number of oocytes and higher-quality embryos. It also improved the endometrium and increased the level of VEGF gene expression. By enhancing the fresh embryo transfer rate in a minimal ovarian stimulation protocol and by improving the clinical pregnancy and ongoing pregnancy rates, the Bushen Yutai recipe could be able to increase fresh embryo transfer and higher-quality embryos.

## Introduction

Although significant advances have been made in the most effective and last line of treatment for infertility, *in vitro* fertilization (IVF) and embryo transfer, the implantation rate remains suboptimal (25%) (1). High-quality embryos and good endometrial receptivity are the key factors for achieving embryo implantation. Currently, with widespread clinical application of the long and short ovarian stimulation protocols, the drawbacks of these protocols are gradually being revealed (2). Ovarian hyperstimulation syndrome (OHSS) is a common complication associated with these protocols, with an occurrence rate of 10–30% (3); and clinical manifestations include pleural effusion, ascites (3), liver and kidney damage, respiratory dysfunction, and other systemic diseases (4). Therefore, to avoid OHSS, the new mild ovarian stimulation protocols are gradually gaining popularity and being widely recognized and accepted by both doctors and patients (5). Mild ovarian stimulation protocols do not require down-regulation with gonadotropin-releasing hormone agonist (GnRHa), and the administered gonadotropin dosages are significantly reduced (6). The total dose of gonadotropin is one-third that of the standard long protocol, thereby significantly reducing the risk of ovarian hyperstimulation, post-operative bleeding, and estrogen-related symptoms. Hence, these are the major advantages of mild ovarian stimulation—less risks, less costs, and less discomfort for patients undergoing assisted reproduction treatment.

However, the use of clomiphene citrate (CC) in mild ovarian stimulation is problematic. In mild stimulation protocols, the administration of CC is routinely used to inhibit the luteinizing hormone (LH) peak and inhibit premature ovulation. But CC has an antiestrogenic effect, which could exert a detrimental effect on the endometrial receptivity. Therefore, it is often the case that most patients cannot receive fresh embryo transfer in mild ovarian stimulation cycles. Embryos have to be vitrified for a warmed-up transfer in a subsequent cycle. The risks of vitrification to long-term embryonic development and infant health are still unclear and remain a serious concern. Additional vitrified–warmed transfer procedures prolong the treatment schedule and add more costs to medical fees. As such, mild ovarian stimulation protocols are worthy of further investigations, as these aim to increase the chances of fresh embryo transfer while maintaining pregnancy rate at a considerately high level. A patient-friendly, cost-effective, and efficient mild ovarian stimulation protocol remains to be optimized. Traditional Chinese medicine (TCM) has been increasingly and widely utilized by women for the treatment of subfertility and has proven to have beneficial therapeutic effects (7). Hence in this study, the effects of Bushen Yutai recipe on the endometrial receptivity of patients undergoing the mild ovarian stimulation protocol were investigated.

## Materials and Methods

### Subjects

This study was carried out in the Assisted Reproductive Center of our Hospital affiliated to Shanghai University of Traditional Chinese Medicine. Two hundred nineteen infertile patients were randomly divided into two groups (the trial registration number: ChiCTR1800016374), the experimental group with patients being administered the Bushen Yutai recipe and the control group with patients not administered the herbal recipe during mild ovarian stimulation IVF treatment. Randomization was achieved through randomized numbers generated by the SAS 9.13 software. The randomized allocation of patients was approved by the ethics committee of our hospital. All participants fulfilled the following criteria: patients were normal responders aged 25–45 years, with basic follicle-stimulating hormone levels of < 10 IU/mL and normal body mass index (18–25 kg/m^2^). All patients were non-smokers and non-alcoholics. The male partners of all patients had normal semen parameters. Both husbands and wives were properly informed before signing the consent forms.

### Mild Stimulation, IVF, and Embryo Transfer

Mild ovarian stimulation commenced on the third day of the menstrual cycle and included oral administration of CC (Fertilan, Cyporus) 25 mg daily, together with intramuscular injection of 150 IU human menopausal gonadotropin (Lizhu Pharmaceutical, China). When at least one follicle reached 18 mL in diameter, or when serum estradiol reached 150 pg/mL per dominant follicle, ultrasound-guided transvaginal oocyte retrieval was performed at 34–36 h after 0.1 mg of GnRHa (Diphereline, France) was subcutaneously injected together with intramuscular injection of 2,000 IU human chorionic gonadotropin (hCG) (Lizhu Pharmaceutical, China). Fertilization of the oocytes was achieved with conventional IVF. The oocytes were examined for fertilization status at 16–18 h after fertilization. Zygotes with two pronuclei were cultured for 48 h in G1 medium (Vitrolife, Sweden). Subsequently, the embryos were examined at the cleavage stage on day 3 after oocyte pickup.

On the hCG triggering day, fresh embryo transfer would be performed if the patients met the following criteria: (1) endometrial thickness was more than 8 mm; (2) endometrium morphology pattern was A or B, and endometrial blood flow was confirmed by positive ultrasound scanning; (3) the number of high-quality embryos on day 3 was more than two; (4) patients had no risk of ovarian hyperstimulation syndrome. The vaginal progesterone gel (Crynone, Merk-serono, Germany) was utilized for luteal phase support. At 14 days after embryo transfer, pregnancy testing was performed by measuring blood hCG levels. After another additional 14 days, transvaginal ultrasound scanning was utilized to detect gestational sac and fetal heartbeat. Clinical pregnancy of patients was confirmed only if both tests were positive.

### Herbal Medicine Administration

In this study, 219 patients were randomly allocated into the TCM experimental group and the control group. Eight patients withdrew from the study. The Bushen Yutai recipe was composed of 10 herbs, including Bushen components, yiqi components, and huoxue components. The herbal components are listed in Table 1.

**Table 1:** The composition of herbal recipes.

**Herbal recipes**		**Herbs**
Bushen Yutai recipe	Bushen recipe	Prepared *rehmannia* root (15 g), *Fructus ligustri lucidi* (6 g), Semen cuscutae (6 g), *Fructus psoraleae* (9 g), *Radix ophiopogonis* (6 g)
	Yiqi recipe	*Astragalus membranaceus* (15 g), Root of hairy asiabell (9 g)
	Huoxue recipe	*Angelica slinensis* (9 g), The root of red-rooted salvia (6 g), *Rhizoma cyperi* (6 g)

**Figure 1:**
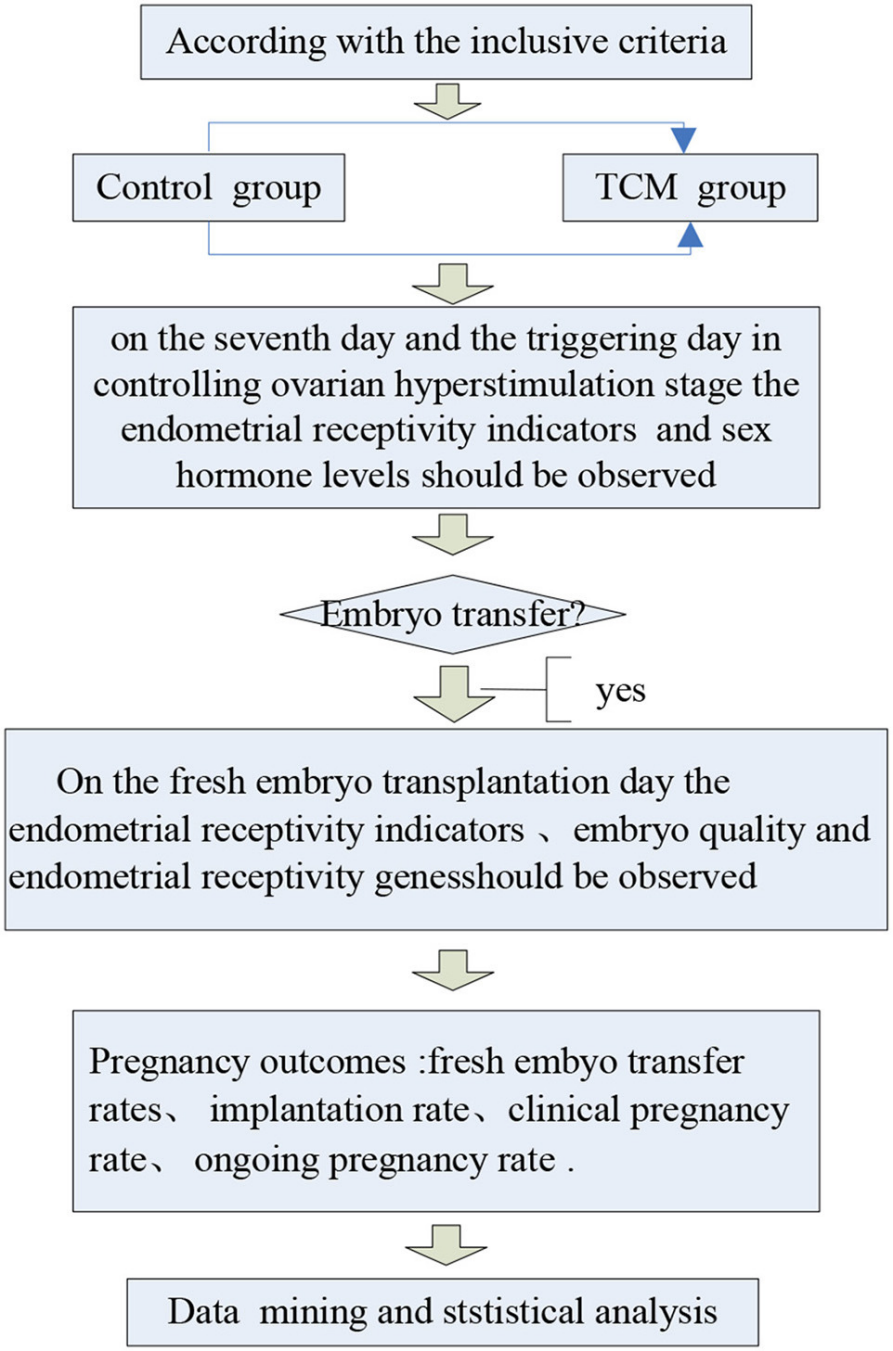
Flowchart of the study to evaluate TCM herbs. Our doctors and researchers had explained the objectives of this study, as well as the procedure and interests of the participants.

The average age, duration of infertility, body mass index, basal FSH level, and basic antral follicle number of patients in the control and experimental groups were comparable, as shown in Tables 2, 3. A flowchart of the study is presented in Figure 1.

**Table 2:** Comparison of the general parameters between the two groups ($\bar{\text{x}}\;\pm$ S).

**Group**	**n**	**Average age (years)**	**Duration of infertility (years)**	**BMI (kg/m^**2**^)**
Control group	106	33.6 ± 4.0	3.2 ± 1.2	21.0 ± 1.7
TCM group	105	32.7 ± 4.6	2.9 ± 1.2	21.1 ± 1.9
*P*		0.1	0.1	0.9

^*^P < 0.05, the difference was statistically significant.

**Table 3:** Comparison of basal sex hormone levels and AFC between the two groups ($\bar{\text{x}}\;\pm$ S).

**Group**	**n**	**FSH (mIU/mL)**	**LH (mIU/mL)**	**Estrodiol (pg/mL)**	**AFC (n)**
Control group	106	6.62 ± 1.89	4.46 ± 2.30	48.08 ± 17.39	0.65 ± 0.36
TCM group	105	6.74 ± 1.64	4.82 ± 2.39	45.69 ± 14.97	0.62 ± 0.43
*P*		0.64	0.27	0.29	0.44

^*^P < 0.05, the difference was statistically significant.

### RNA Isolation, Reverse Transcription, and Real-Time Polymerase Chain Reaction

RNA was extracted or isolated by using Trizol Reagent (Invitrogen, Carlsbad, CA, USA) according to the manufacturer's instructions. Total RNA was measured by spectrophotometry at 260 nm, and 1 mg of total DNase-treated RNA per sample was used for reverse transcription by a SuperScript III kit (Invitrogen), following the manufacturer's instructions. Specific primers and probes were purchased from Applied Biosystems (Life Technologies, USA). Quantitative real-time polymerase chain reaction (qRT-PCR) analysis was performed with the 7,300 Real-Time PCR System (Applied Biosystems, USA), using SYBR Green (Life Technologies). The primer sequences of gene markers utilized for qRT-PCR analysis are shown in Table 4, with GAPDH (glyceraldehyde 3-phosphate dehydrogenase) being utilized as the endogenous reference control gene. The following amplification parameters were utilized for the qRT-PCR analyses: 15 min at 95°C, and 40 cycles of 10 s at 95°C, followed by 30 s at 60°C. The 2-ΔΔCt method was used to compute the relative cycle threshold (Ct) values for each gene, which were then normalized against the endogenous GAPDH gene expression. Altogether, there were three experimental replicates for each gene analyzed by qRT-PCR.

**Table 4:** Comparison of primer sequences between the two groups.

**Gene markers**	**F-primer sequences**	**R-primer sequences**
IL-8	TCCAAACCTTTCCACCCCAA	CCACAACCCTCTGCACCCA
IL-6	AAGCAGCAAAGAGGCACTGG	TGGCATTTGTGGTTGGGTCA
LIF	CCCAACGTGACGGACTTCCC	AGGCCTCGCAGGATGTCG
VEGF	CAGCTACTGCCATCCAATCGAG	CCTATGTGCTGGCCTTGGTG
H0XA10	ACGGCAAAGAGTGGTCGGAA	TAAAGTTGGCTGTGAGCTCCC

### Statistical Analysis

Statistical analysis was performed using the SPSS 21 statistical software. Continuous data were presented as the mean ± SD and compared using the one-way analysis of variance test. Differences between ratios and percentage values were analyzed using the χ^2^ test. *P* < 0.05 was considered to be statistically significant.

## Results

### Comparison of Embryo Quality Between the Two Groups

**Figure 2:**
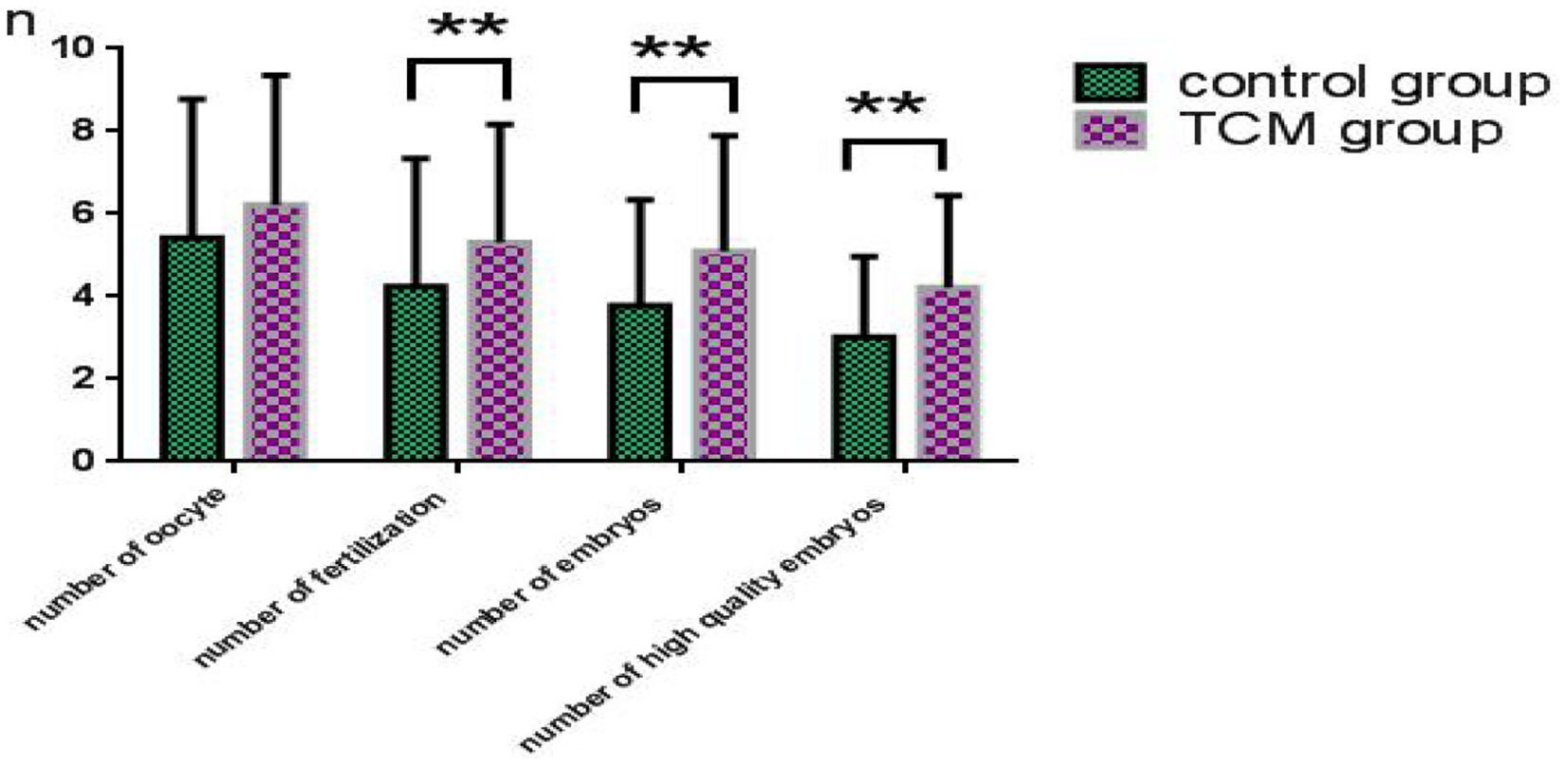
TCM treatment yielded more good quality embryos. **Means statistically significant much.

The data on embryo quality in the control and experimental groups are presented in Figure 2. The number of oocytes in the TCM experimental group (6.2 ± 3.1) was higher than that of the control group (5.4 ± 3.4), even though the difference was not statistically significant (*P* > 0.05). The fertilization rate in the TCM experimental group was significantly higher than that of the control group, and so was the number of embryos in the TCM experimental group (5.1 ± 2.8) vs. the control group (3.8 ± 2.6) (*P* < 0.01 in both cases). Additionally, the number of high-quality embryos (4.2 ± 2.2) was also significantly higher than that of the control group (3.0 ± 2.0) (*P* < 0.01).

### Comparison of Sex Hormone Levels Between the Two Groups on the hCG Triggering Day

The data on the sex hormone levels of the control and experimental groups are presented in Table 5. Estradiol level in the TCM experimental group (2510.8 ± 1135.5 pg/mL) was significantly higher than that of the control group (2098.6 ± 1215.5 pg/mL), (*P* < 0.05). The LH level in the TCM experimental group (7.9 ± 3.8 mIU/mL) was lower than that in the control group (8.7 ± 6.3 mIU/mL). The progesterone (P) level in the TCM experimental group (1.3 ± 0.8 ng/mL) was higher than that in the control group (1.2 ± 0.9 ng/mL), but the difference was not statistically significant (*P* > 0.05).

**Table 5:** Comparison of sex hormone levels between the two groups ($\bar{\text{x}}\;\pm$ S) on the hCG triggering day.

**Group**	**n**	**LH (mIU/mL)**	**Estrodiol (pg/mL)**	**Progesterone (ng/mL)**
Control group	106	8.7 ± 6.3	2098.6 ± 1215.5	1.2 ± 0.9
TCM group	105	7.9 ± 3.8	2510.8 ± 1135.5	1.3 ± 0.8
*P*		0.3	0.0^*^	0.8

* *Means Statistically significant*.

### Comparison of Endometrial Receptivity Indicators

**Figure 3:**
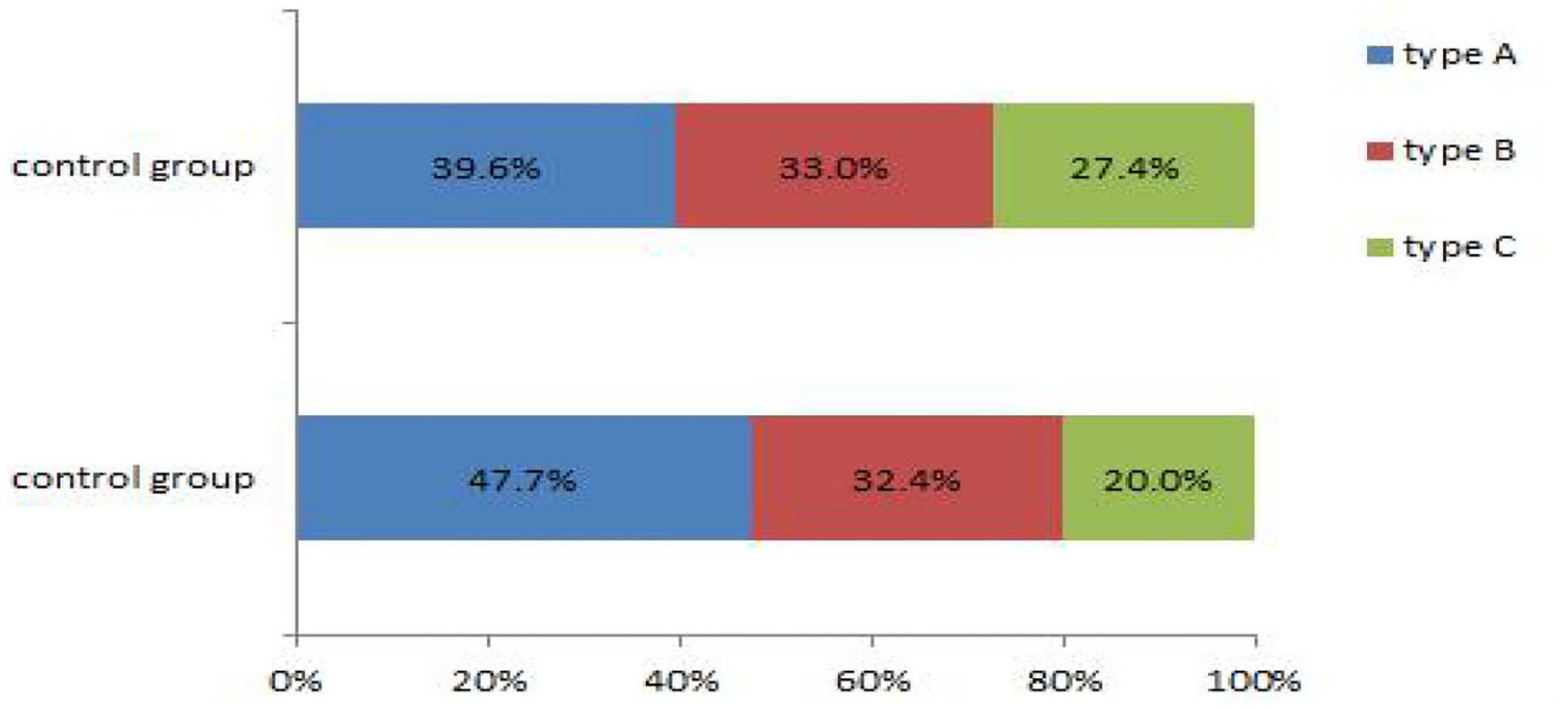
More positive blood flow was seen in the TCM experimental group.

**Figure 4:**
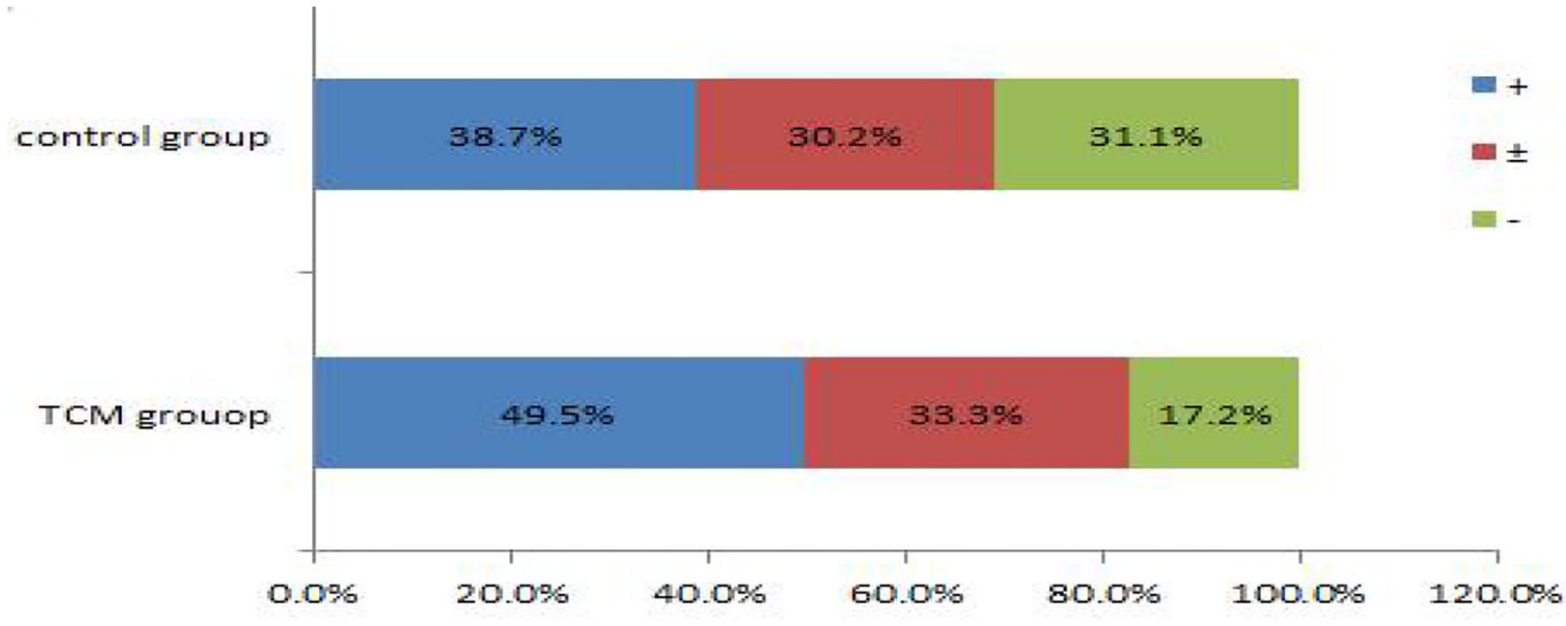
Comparison of endometrial blood flow velocities on the hCG triggering day.

The data on the endometrial receptivity indicators of the control and experimental groups are presented in Table 6 and Figures 3, 4. On the hCG triggering day, the endometrial thickness of the TCM experimental group (8.7 ± 2.6 mm) was higher than that of the control group (8.3 ± 2.7 mm), but the difference was not statistically significant (*P* > 0.05). There were altogether 42 cases (39.6%) of type A endometrium and 35 cases (33.0%) of type B endometrium, and 29 cases (27.4%) of type C endometrium in the control group. Within the TCM experimental group, there were 50 cases (47.6%) of type A endometrium, 34 cases (32.4%) of type B endometrium, and 21 cases (20%) of type C endometrium. The rates of the good (type A) and the fairly good (type B) morphology endometrium was not significantly different (*P* > 0.05). However, more patients had positive endometrium blood fold under ultrasound scanning (49.5 vs. 38.7%)(*P* < 0.01). In the control group, the endometrial blood flow parameters proactive inhibition (PI) (1.4 ± 0.7) and retroactive inhibition (RI) (0.7 ± 0.2) were higher than that of the TCM experimental group PI (1.1 ± 0.8) and RI (0.6 ± 0.5), and the difference was statistically significant (*P* < 0.05). Similar results were also observed with embryo transfer 3 days later.

**Table 6:** Comparison of the endometrial blood flow parameters PI and RI between the two groups ($\bar{\text{x}}\;\pm$ S) on the hCG triggering day.

**Group**	**n**	**PI**	**RI**
Control group	106	1.4 ± 0.7	0.7 ± 0.2
TCM experimental group	105	1.1 ± 0.8	0.6 ± 0.5
*P*		0.0^**^	0.0^**^

** *Means Statistically significant much*.

### Comparison of the Expression Levels of Endometrial Receptivity Genes Between the Two Groups on the Day of Fresh Embryo Transfer

**Figure 5:**
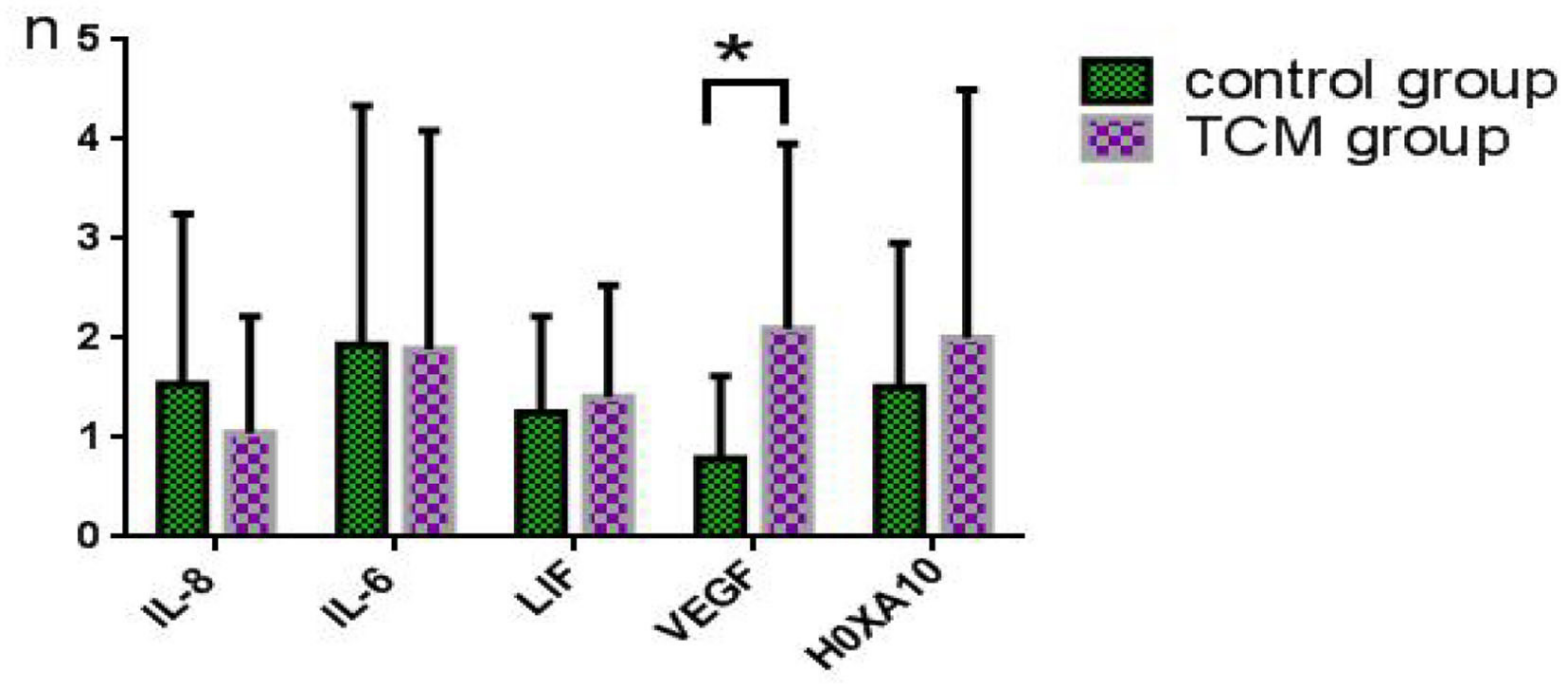
Comparison of the expression levels of endometrial receptivity genes between the two groups on the day of fresh embryo transfer. *Means statistically significant.

The data on endometrial receptivity gene expression of the control and experimental groups are presented in Table 7 and Figure 5. The relative expression levels of IL-8, IL6, LIF, and HOXA10 were similar between the TCM experimental group and control group (*P* > 0.05). The relative expression level of the vascular endothelial growth factor (VEGF) gene in the TCM experiment group was (0.8 ± 0.8), which was significantly higher than that in the control group (2.1 ± 1.9) (*P* < 0.05).

**Table 7:** Comparison of the relative expression levels of VEGF between the two groups ($\bar{\text{x}}\;\pm$ S).

**Group**	**n**	**IL-8**	**IL-6**	**LIF**	**VEGF**	**H0XA10**
Control	14	1.5 ± 1.7	1.9 ± 2.4	1.3 ± 1.0	0.8 ± 0.8	1.5 ± 1.4
TCM	22	1.1 ± 1.1	1.9 ± 2.2	1.4 ± 1.1	2.1 ± 1.9	2.0 ± 2.5
*t*		1.0	0.7	0.4	1.5	0.7
*P*		0.3	0.5	0.7	0.0^*^	0.5

* *Means Statistically significant*.

### Comparison of Pregnancy Outcomes Between the Two Groups

The data on the pregnancy outcomes of the control and experimental groups are presented in Table 8. Thirty-five patients (33.0%) in the control group met good endometrium criteria and therefore received fresh embryo transfer, whereas in the TCM experimental group, there were 48 patients (45.7%) with satisfactory endometrium who received fresh embryo transfer. Therefore, more patients in the TCM experimental group could receive fresh embryo transfer than the control group (*P* < 0.05). Unfortunately, there was one case in the control group and three cases in the TCM experimental group who canceled fresh embryo transfer due to catching a cold with high body temperature. Finally, 34 patients in the control group and 45 patients in the TCM experimental group received fresh embryos. The clinical pregnancy rate (38.2%) in the control group was lower than that in the TCM experimental group (40%) (*P* > 0.05). The embryo implantation rate was not significantly different either, with 20.6% for the control group vs. 24.4% for the TCM experimental group, respectively (*P* > 0.05). There were two pregnancy losses (15.4%) in the control group. However, miscarriage did not occur in the TCM experimental group.

**Table 8:** Comparison of pregnancy outcomes between the two groups (%).

**Group**	**n**	**Fresh embryo transfer rate (%)**	**Clinical pregnancy rate (%)**	**Implantation rate (%)**	**Miscarriage rate (%)**
Control	34	35 (33.0%)	35.3% (12/34)	20.6% (14/68)	15.4% (2/12)
TCM	45	48 (45.7%)	40.0% (18/45)	24.4% (22/90)	0% (1/18)
*P*		0.0^*^	0.4	0.3	0.2

* *Means Statistically significant*.

## Discussion

This study was a randomized controlled clinical trial to evaluate the effects of the Bushen Yutai recipe and its three components on the endometrial receptivity and ovarian responses of patients subjected to a particular mild ovarian stimulation regimen. Embryo implantation will be successful only if it occurs within a limited time period known as “the window of implantation,” when the blastocyst is implantation-competent, and the endometrium is receptive (8). Additionally, several studies have shown that pregnancy will occur only when the endometrial thickness falls within a particular range of values (9, 10). On the hCG triggering day, the endometrial thickness of the TCM experimental group was higher than that of the control group, which indicated that the Bushen Yutai herbal recipe increased endometrium thickness.

As an adequate blood supply to the embryo is required for normal fetal growth, angiogenesis plays a key role during implantation (11). Dysregulated endometrial angiogenesis has been reported to lead to infertility (12). VEGF within the endometrial secretion could promote new angiogenesis within the endometrium and is known to be highly expressed during the early phase of endometrial proliferation (13). In particular, VEGF may be the most important growth factor for the development and maintenance of blood vessels (14). Our study showed that expression levels of VEGF in the TCM experimental group were significantly higher than those of the control group. VEGF could form new blood vessel networks, improve blood perfusion, and increase the implantation rate. Our data also showed that the TCM experimental group blood flow parameters PI and RI were significantly lower than those of the control group. Additionally, we also found that positive blood flow was more frequently observed in the TCM experimental group. All these thus proved that the Bushen Yutai recipe increased blood perfusion, improved the microenvironment and endometrial receptivity, and was conducive to embryo implantation.

Many TCM remedies are formulated with diverse species of herbs to increase effectiveness and reduce adverse effects through interaction with multiple molecular targets and biological pathways (15). The biological mechanisms underlying the observed beneficial effects of the Bushen Yutai recipe and its three components remain unclear. The results of this study, however, demonstrated that the positive effects might be achieved through multitarget, multisystem regulation to improve the body's endocrine levels, thereby increasing the estradiol levels and leading to increased number of oocytes and high-quality embryos. It could also involve upregulating VEGF gene expression levels in the endometrial exfoliated cells, as well as reduction of the blood flow resistance, which in turn improved the patient's endometrial receptivity. The Bushen Yutai recipe could increase the likelihood of fresh embryo transfer in patients undergoing the mild ovarian stimulation protocol, in which fresh embryo transfer is seldom performed. This is of utmost interest to patients undergoing mild ovarian stimulation because this would decrease the number of required procedures such as embryo vitrification and warming, as well as reduce the number of hospital visits and lessen the risks of cryopreservation.

## Conclusion

The Bushen Yutai herbal recipe improved the endometrial receptivity by increasing endometrial blood blow. For patients undergoing mild ovarian stimulation whereby fresh embryo transfer is rarely performed, this Traditional Chinese Medicine herbal formula increased the likelihood of fresh embryo transfer in a single treatment cycle.

## Data Availability Statement

All datasets generated for this study are included in the article/supplementary material.

## Ethics Statement

The studies involving human participants were reviewed and approved by IRB of shuguang hospital affiliated with shanghai university of TCM. The patients/participants provided their written informed consent to participate in this study. Written informed consent was obtained from the individual(s) for the publication of any potentially identifiable images or data included in this article.

## Author Contributions

XJ: mainly designed experiments, clinical and experimental studies, statistical data, analysis data, and writing papers. YH: an expert in traditional Chinese medicine, has given a lot of help to the differentiation and classification of clinical Chinese medicine, especially the tongue and pulse, so that the clinical research can be carried out smoothly. ZX: the experimental research methods and steps to play a supervisory role, to ensure the accuracy of the data. TG and ZW: provided funding for the project, guided and corrected all aspects of the project, so as to complete the project.

## Conflict of Interest

The authors declare that the research was conducted in the absence of any commercial or financial relationships that could be construed as a potential conflict of interest.
